# Hypofractionation Utilisation in Radiation Therapy: A Regional Department Evaluation

**DOI:** 10.1002/jmrs.857

**Published:** 2025-02-25

**Authors:** Cyrena Tabet, Amy Brown, Catriona Hargrave, Savannah Brown

**Affiliations:** ^1^ School of Clinical Sciences, Faculty of Health Queensland University of Technology (QUT) Brisbane Queensland Australia; ^2^ Radiation Oncology Princess Alexandra Hospital – Raymond Tce Campus Brisbane Queensland Australia; ^3^ Radiation Therapy Townsville Hospital and Health Service Townsville Queensland Australia

**Keywords:** Aboriginal and Torres Strait islander, breast, hypofractionated radiotherapy, modality, out‐of‐town, palliative, prostate, regional, rural, ultrafractionated radiotherapy

## Abstract

**Introduction:**

There has been an uptake in hypofractionation radiotherapy schedules (> 2.45 Gy per fraction) worldwide over the last decade. The aim of this paper was to evaluate the change in fractionation schedules for patients undergoing radiotherapy in regional Queensland. The influence of treatment site, intent and patient social circumstances was assessed, identifying any current gaps in practice.

**Methods:**

This retrospective clinical audit, included patients who underwent radiotherapy in 2012, 2019 and 2022 at a large regional department. This allowed a 10‐year analysis and an evaluation of any impact of COVID‐19. Demographic data and treatment information was collected and analysed using descriptive statistics.

**Results:**

There was a notable trend favouring hypofractionation for patients treated for breast and prostate cancer. In 2012, 62.7% of breast cancer patients were treated with conventional fractionation and 37.3% were treated with hypofractionation, versus 2.4% and 92.1%, respectively, in 2022. Prostate cancer fractionation changed from 99.4% of patients treated with conventional fractionation and 0.6% with hypofractionation in 2012 to 23.2% and 74.1%, respectively, in 2022. The standard of care also shifted for palliative intent, with lung, brain and bone metastases in 2022 being treated with increased hypofractionated and ultra‐hypofractionated radiotherapy (> 5 Gy per fraction). This coincides with more complex and modulated treatments being readily available, such as stereotactic radiotherapy and volumetric modulated arc therapy. Hypofractionated treatments, however, were not influenced by the social factors of patients, having no distinct relationship with Indigenous status, age and patients' distance to treatment.

**Conclusion:**

This study has validated the increase in hypofractionated treatments over a range of cancer sites and treatment intents, with increased treatment complexity. This has a direct impact on both departmental resources and patient‐centred care, offering value‐based radiotherapy.

## Graphical abstract

This study has validated the increase of hypo fractionated treatments over a range of cancer sites and treatment intents, with increased treatment complexity over a decade (2012, 2019, 2022). This has a direct impact on both the departmental resources and patient‐centred care, offering value‐based radiotherapy. Patient demographics, such as distance from treatment facilities and First Nation patients, did not significantly impact the prescription of HF, instead, indicating that decision‐making by radiation oncologists at our centre may be primarily driven by clinical factors.

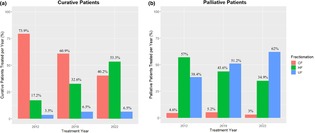

## 1 Introduction

Hypofractionated radiotherapy (HFRT) is a fractionation scheme that administers a biologically equivalent dose in fewer fractions, with more dose per fraction. With new theoretical data that endorsed the use of HFRT, multiple clinical trials have emerged across curative and palliative contexts, effectively altering the standard of care [1, 2]. The palliative patient cohort has similarly seen a large adoption of HFRT and ultra‐fractionated radiotherapy (UFRT), while introducing more complex treatments for these patients.

For breast treatment, the fractionation regimen has evolved from 50 Gy in 25# to 40 Gy in 15#, with recent trial data promoting the adoption of 26 Gy in 5# [3]. Similarly, prostate treatment fractionations have shifted from 78/80 Gy in 39–45# to 60 Gy in 20# [4], with emerging data for 5# regimens [4]. Recently, this has been clinically implemented with the emergence of online adaptive technology, as it allows for greater precision of the visible tumour and can account for daily positional changes [5]. The implementation of HFRT for breast and prostate cancer is evidence‐based, while also promoting convenience, less travel for out‐of‐town patients and positive patient experiences. In addition to this, HFRT provides an increase in efficiency within the department [6].

Clinical evidence has also supported the integration of HFRT and UFRT treatments for palliative patients. For patients with a palliative prognosis, especially near the end of life, longer treatment decreases the chance that the patient will receive the full treatment and may not provide any additional clinical benefit [7, 8]. Single‐fraction radiotherapy (RT) for palliative patients can be considered highly effective in providing quick pain relief and streamlining the patient's treatment to a single hospital visit [9].

The rise in HFRT utilisation in Australia has been documented and is supported by the five Choosing Wisely guidelines for radiation oncology, two of which advocate for the consideration of shorter fractionation schedules for breast cancer patients, and single‐fraction schedules for non‐complex bone metastases [10]. The uptake of HFRT and UFRT, in keeping with emerging clinical evidence, is of high importance in achieving value‐based health care and has been recognised as a priority in New South Wales [11].

Uptake of HFRT and UFRT in Australia has varied across different cancer patient cohorts requiring radiation therapy. Multiple studies found that the uptake of RT for palliative patients in Australia was robust but slow [12]. The utilisation of HF in the Australian Capital Territory between 2006 and 2018 for breast cancer patients increased from 12.1% to 56.6% [13]. A Victorian audit corroborated this, with an increase from 35% in 2012 to 66% in 2017 [14]. Several reviews have identified an increase in HFRT prostate treatments in the last few years, with approximately 40% of prostate patients in Australia being treated with HFRT in 2018 and 2019 [15, 16]. Throughout Australia, there are still some regions and RT services that have delayed uptake of HF in the radical setting. A national retrospective audit discovered the proportion of HF patients treated in different local health districts for breast and prostate cancer ranged from 24.9% to 100% [15]. Internationally, departments with online adaptive technology such as the MRI Linac have reported higher uptakes of UF, with 71%–80% of prostate patients treated with UFRT regimens in 2020 [17, 18].

The aim of this project was to assess Townsville Cancer Centre's (TCC) patterns of care over three time periods (2012, 2019 and 2022) for multiple treatment sites, treatment intents and patient demographics. As TCC is the largest department in North Queensland, HFRT has a notable impact on working‐age patients and patients who live a significant distance from the department, particularly First Nations patients. This project assessed the adoption of HFRT since 2012 in a regional setting, informing and identifying current gaps in this practice. Secondary objectives assessed the influence patient characteristics, such as social circumstances, have on the prescription of HFRT, as well as the changes in complexity and treatment techniques.

## 2 Methods

### 2.1 Patient Data

The institutions providing ethics approval was the Human Research Ethics Committee (HREC) and Queensland University of Technology (QUT) granted to conduct this retrospective cross‐sectional audit (HREC/2022/QTHS/89999), including a waiver of consent. Patient inclusion criteria consisted of patients who had completed a course of RT in 2012, 2019 and 2022. This patient cohort allowed a 10‐year analysis (2012–2022) to be conducted, whilst also exploring any potential impact from COVID‐19 by using data captured prior to the pandemic reaching Australia.

The data were extracted from MOSAIQ (Elekta, Stockholm, Sweden). MOSAIQ was unable to extract the data prior to April 2012 due to an upgrade changing data characteristics, so data from April 2012 to March 2013 were used to represent the nearest full calendar year. The initial spreadsheet included patient demographics and relevant treatment data, as outlined in Table 1.

**TABLE 1:** Explanation of the variables in the dataset.

Variable	Category groups	Description
Gender	Female/male	—
First Nations status	Aboriginal and Torres Strait islander, Aboriginal not Torres Strait islander, Torres Strait islander not Aboriginal, not Aboriginal or Torres Strait islander, Unknown/not defined	—
Age in years	Numerical	Age of patient when undergoing treatment
Suburb and postcode	Nominal: suburb name and postcode	Collected from patient place of residence
Radiation oncologist (RO)	Nominal: 1–11	Each RO is associated with a number for deidentification purposes
ICD code	Nominal: e.g., C10.8	Cancer diagnosis code according to the International Classification of Diseases [19] (ICD)
ICD description	Nominal	ICD definition of diagnosis
Treatment intent	Unknown, prophylactic, adjuvant, palliative, curative, curative adjuvant, improve survival, neoadjuvant	As indicated in the radiation oncologist's care plan
Course number	Ordinal: first course = 1, second course = 2, etc.	The patient's current course of treatment's number, recognising that some patients have several courses of treatment over a period
Treatment year	2012, 2019, 2022	The year the patient was treated for the course of treatment
Site name	Nominal	Doctors' description of patient's treatment site
Dose and fractions	Numerical	Dose (Gy) prescribed and number of fractions (#)
Treatment technique	2D/Field Defined, 3DCRT, IMRT, VMAT, Electron etc.	Technique used to deliver the treatment, derived from radiation oncologist's prescription
Treatment modality	XRAY 6MV, 6 MeV, Mixed etc.	Beam energy, derived from radiation oncologist's prescription
Prescription status	Approved/pending	Doctor's approval status of the patient's prescription. When treatment is ceased early without completing the prescribed fractionation, it is standard practice to update the prescription to ‘pending’ status.
Field	Static, kV image, step and shoot, CBCT, etc.	Type of treatment field and imaging field
Pattern	Daily, weekly, biweekly	Frequency of treatment, derived from radiation oncologist's prescription
Pattern defined	Everyday/week, specific days	Defines if for daily treatment or a specific interval, derived from radiation oncologist's prescription
Pattern week	Specific day/week, Mon/Thurs	Days during the week patient will receive treatment, derived from radiation oncologist's prescription
Schedule status	SC/OC	SC refers to the start of a course with no previous treatment in the department, which has been captured, and OC refers to the start of a course of treatment where the patient has had a previous course of treatment in the department
Time from prescription to start	Numerical	Number of days calculated from when prescription was approved to the treatment start date

Abbreviations: 2D/Field Defined, 2‐dimensional; 3DCRT, 3‐dimensional conformal radiation therapy; CBCT, cone beam computed tomography; ICD, Internal Classification of Diseases; IMRT, intensity modulated radiation therapy; kV, kilovoltage; MeV, megaelectron volt; MV, megavoltage; VMAT, volumetric modulated arc therapy.

### 2.2 Data Cleaning

The dataset was imported into R studio 4.3.1. To avoid the potential of re‐identifying a single patient from a low‐population rural town via the postcode, treatment details and demographics, the suburb and postcode were transformed to a linear distance (kilometres), calculated from the department to the patient's residing suburb. The suburbs were also categorised by Accessibility/Remoteness Index of Australia Plus (ARIA+) [20]. ARIA+ Remoteness Area Category codes are defined from 0 to 4, with zero representing major cities and four representing very remote areas. However, for some suburbs, the search tool could not identify an ARIA+ code. To mitigate this, postcodes were used, or if that was invalid, neighbouring suburbs were used as substitutions. For suburbs and postcodes that had more than one ARIA+ code, the lower ARIA+ code was used for definition.

Patients that had ±35 days from prescription approval to treatment start data were filtered out, due to MOSAIQ repeating data for certain patients when they had multiple courses during 2012–2022. MOSAIQ exported each treatment field for each course for a patient as a separate row. The data was reformatted so that the treatment fields for each course of treatment were combined in one row, with a new variable added that represented the total number of treatment fields per course. Site name, technique field type and modality were re‐categorised to ensure naming consistency (refer to Table S1).

Patients without dose or fraction information were removed from the dataset. The clean dataset had a sample size of 2628 patients.

### 2.3 Data Transformation

In the era of modulated radiotherapy, several patients are treated with concomitant boosts, which was taken into consideration when defining the fractionation type differently from the standard definition of conventionally fractionated radiotherapy (CFRT). The fractionation type for curative patients was defined, with less than 2.45 Gy defined as CFRT, less than 5 Gy defined as HFRT and greater than 5 Gy defined as UFRT. Palliative intent fractionation was defined differently, due to palliative regimens generally being larger doses per fraction In palliative cases, CFRT was defined as less than 3.1 Gy, HFRT as less than 6 Gy and UFRT as greater than 6 Gy. The specific prescriptions used across the different treatment sites at TCC were used (detailed information in Table S2).

For curative patients, the World Health Organization's International Classification of Diseases (ICD) codes were used to define general treatment sites [19]. For palliative patients, the ICD code represented the initial or primary diagnosis. Therefore, the site name was used to rename the treatment site to bone metastases, brain metastases, lung metastases, pelvis palliative, breast/CW palliative or abdominal palliative. Various palliative anatomical sites where the site name did not facilitate treatment site renaming using these criteria were renamed to ‘local control/advanced disease’.

Distance to treatment was grouped into five categories: < 25, < 50, < 100, < 500, < 1000 and ≥ 1000 km. Additionally, age was categorised to represent patients that are of working age. This study used the Organisation for Economic Co‐operation and Development (OECD) definition of working age (15–64), noting that paediatric patients are not standardly treated at TCC [21].

### 2.4 Data Analysis

To determine the relationship between fractionation type and patient/treatment factors, bar plots, counts and percentages were developed and then analysed. Fractionation type, treatment year and treatment site were grouped to analyse the change in fractionation for breast, prostate, palliative sites, curative sites, bone metastases, lung metastases and brain metastases. Grouped bar plots were created for each treatment site. Similarly, this was performed for demographic data, grouping First Nations status, ARIA+ code, distance to treatment and working‐age patients. Lastly, grouped bar plots were created to assess the relationship between the change in treatment modality over the 10 years for each requested treatment site.

## 3 Results

**FIGURE 1:**
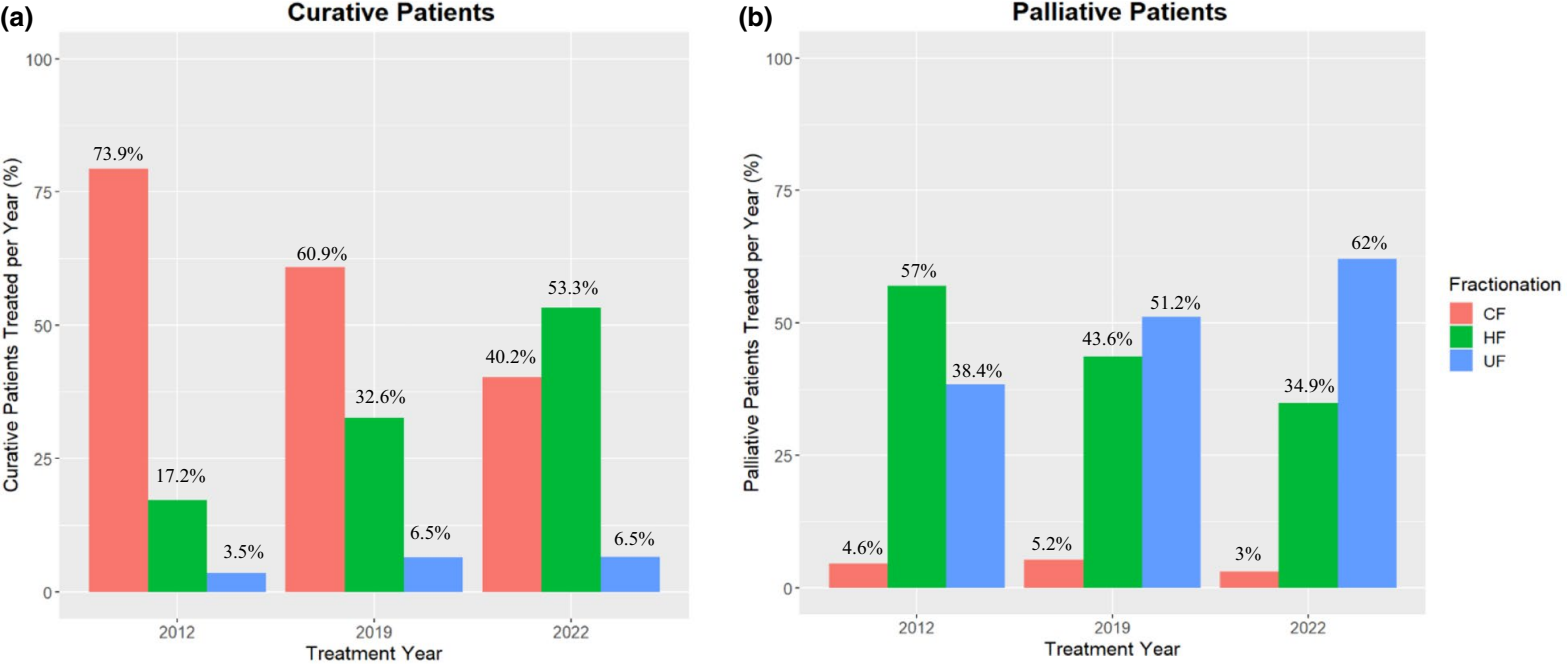
Relationship between treatment year, fractionation type and treatment intent; curative (a) and palliative (b). CF, conventional fractionation; HF, hypofractionation; UF, ultrafractionation.

This study included 2628 patients: 1060 in 2012, 778 in 2019 and 790 in 2022, with a median age of 65.7 (18–98) years and 55.5% being male. The mean distance to treatment was 117 km, and 79% of patients resided in an ARIA+ code of two (further of breakdown of patient demographics and characteristics provided in Table S3). For curative patients, there was a steady increase in patients treated with HFRT and a decrease in CFRT. Patients treated with UFRT did not change significantly from 2012 to 2022. On the other hand, palliative patients had a shift towards UFRT, rather than HFRT, with a small portion treated with CFRT over the course of the study (Figure 1).

**FIGURE 2:**
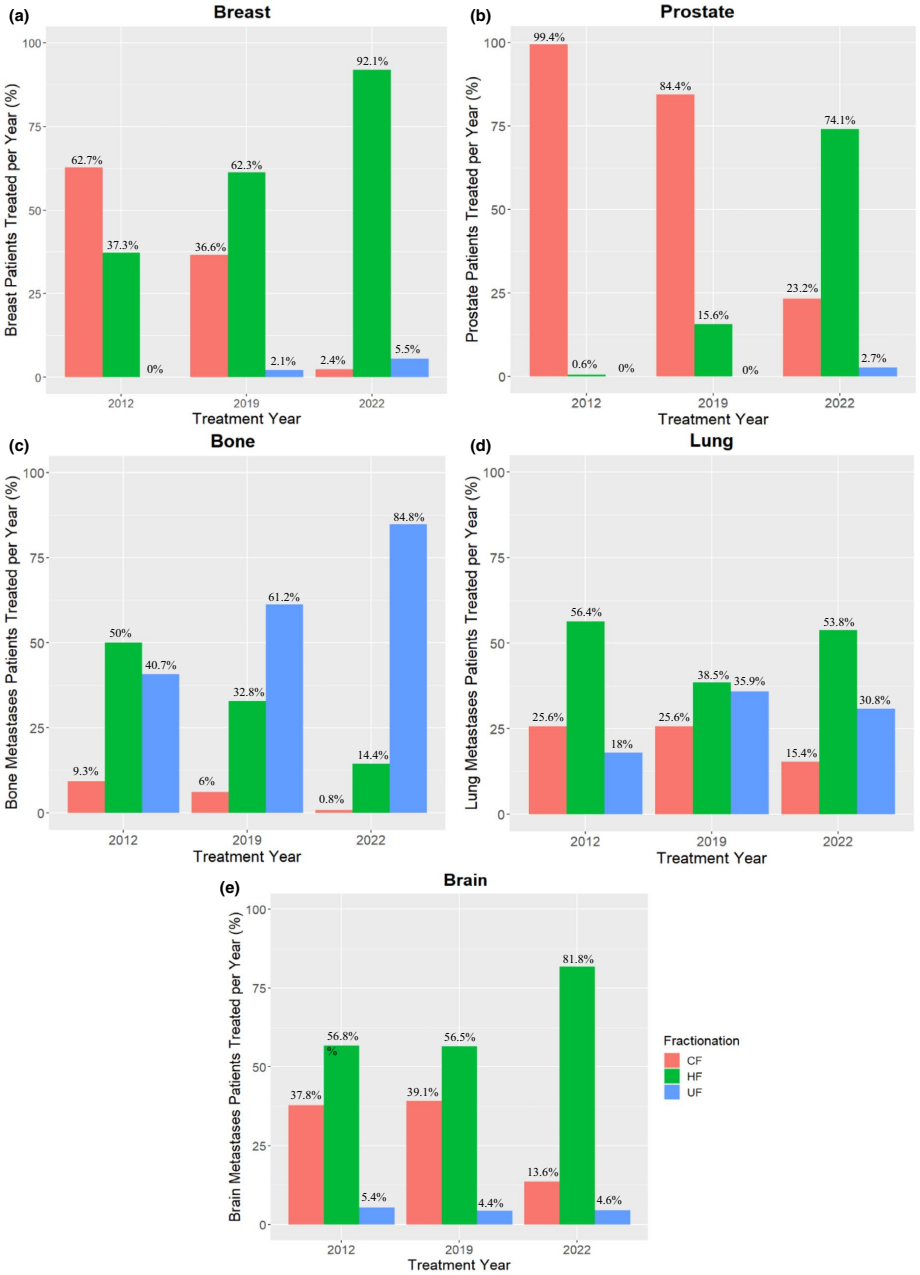
Relationship between treatment year, fractionation type and site of breast (a), prostate (b), bone metastases (c), lung metastases (d) and brain metastases (e). CF, conventional fractionation; HF, hypofractionation; UF, ultrafractionation.

Over the course of the study, breast cancer treatments saw a shift from CFRT to HFRT. In 2019 and 2022, there was a small increase in patients treated with UFRT. For prostate patients, the change in fractionation scheme was prominent after 2019, with a majority of patients treated with HFRT and a small adoption of UFRT in 2022. For bone metastases, the change was primarily between HF and UF treatments, with under 1% of patients being treated with CFRT in 2022. For patients with lung metastases, the trend of UFRT uptake was not clear; however, there was a decrease in CFRT utilisation. For brain metastases, the UFRT regimens remained consistent through the timepoints evaluated in this study. There was a large shift in 2022 from CFRT to HFRT (Figure 2).

**FIGURE 3:**
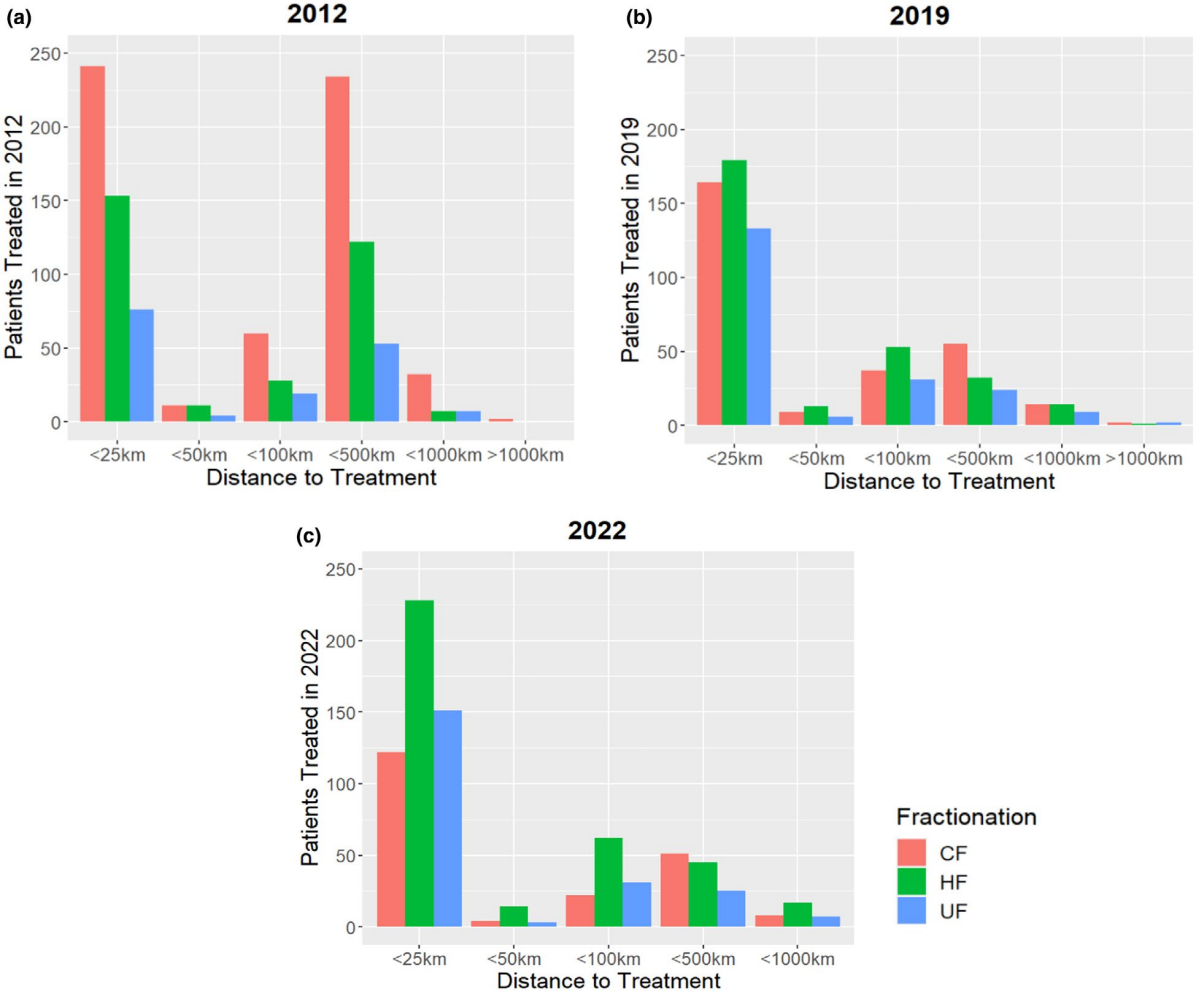
Relationship between fractionation type, distance to treatment and treatment year 2012 (a), 2019 (b) and 2022 (c). CF, conventional fractionation; HF, hypofractionation; UF, ultra‐fractionation.

The relationship between fractionation type and patients' distance to treatment showed no clear trend. From 2012 to 2022, the utilisation of HF increased for all distance cohorts (Figure 3). There was also no clear relationship between ARIA+ code and utilisation of HF (refer to Figure S1).

**FIGURE 4:**
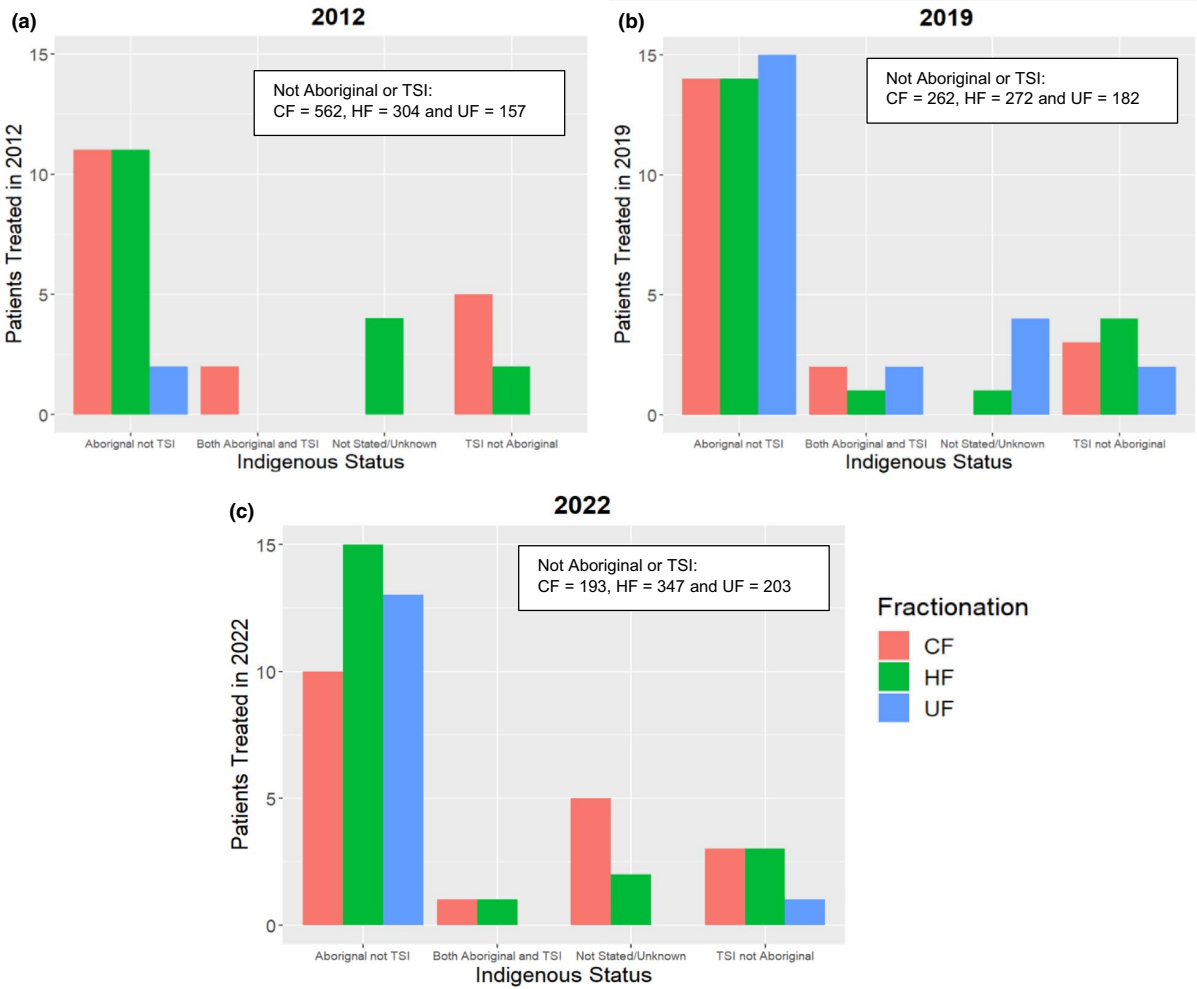
Relationship between fractionation type, First Nations status and treatment year 2012 (a), 2019 (b) and 2022 (c). CF, conventional fractionation; HF, hypofractionation; UF, ultra‐fractionation.

There was no clear trend found with First Nations patients and utilisation of HF (Figure 4).

**FIGURE 5:**
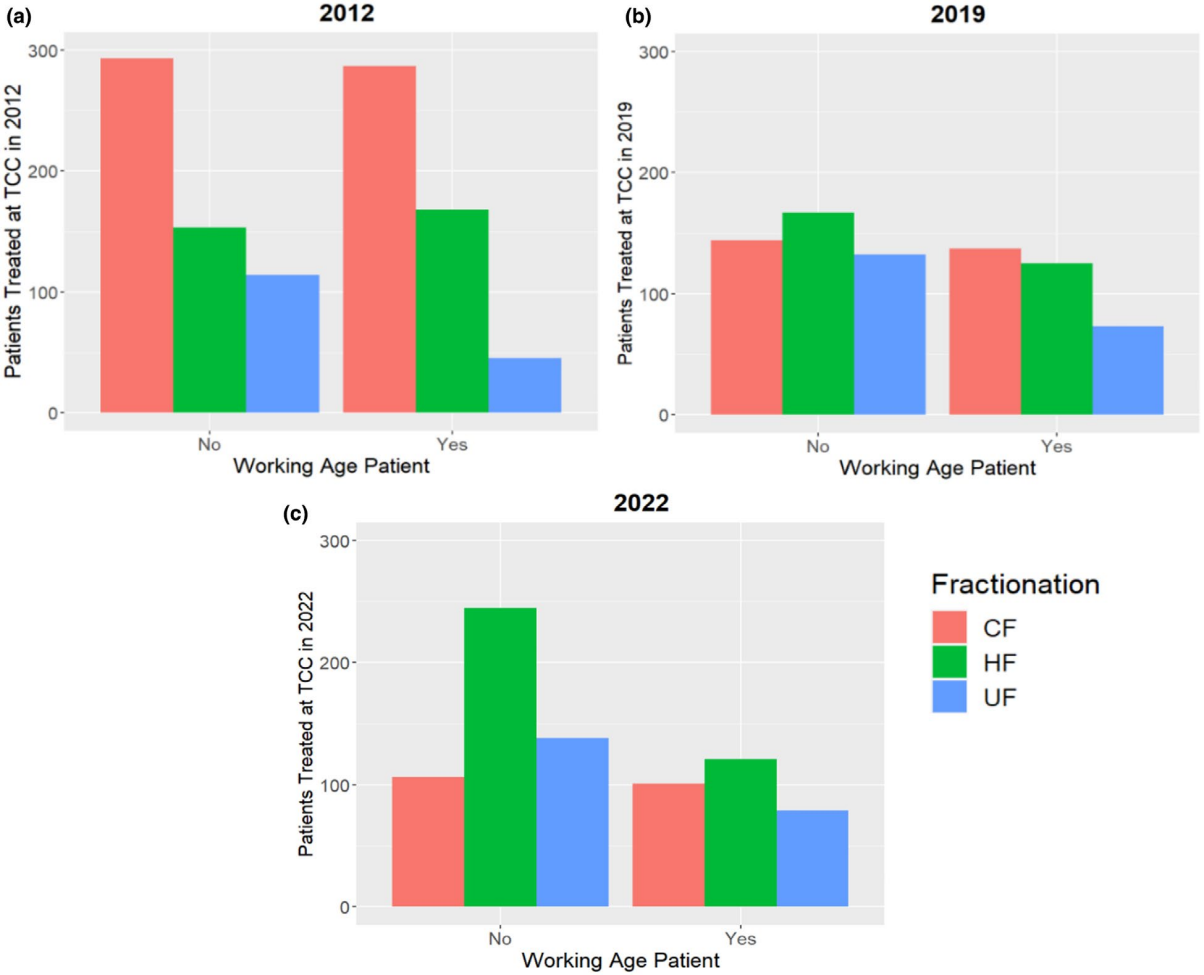
Relationship between fractionation type, working‐age patients and treatment year 2012 (a), 2019 (b) and 2022 (c). CF, conventional fractionation; HF, hypofractionation; UF, ultra‐fractionation; where No = patients > 65 years, Yes = patients < 65 years.

There was no clear trend found with patients and their working‐age status (Yes or No) and utilisation of HF, as there was an uptake of HF in both patient cohorts (Figure 5).

**FIGURE 6:**
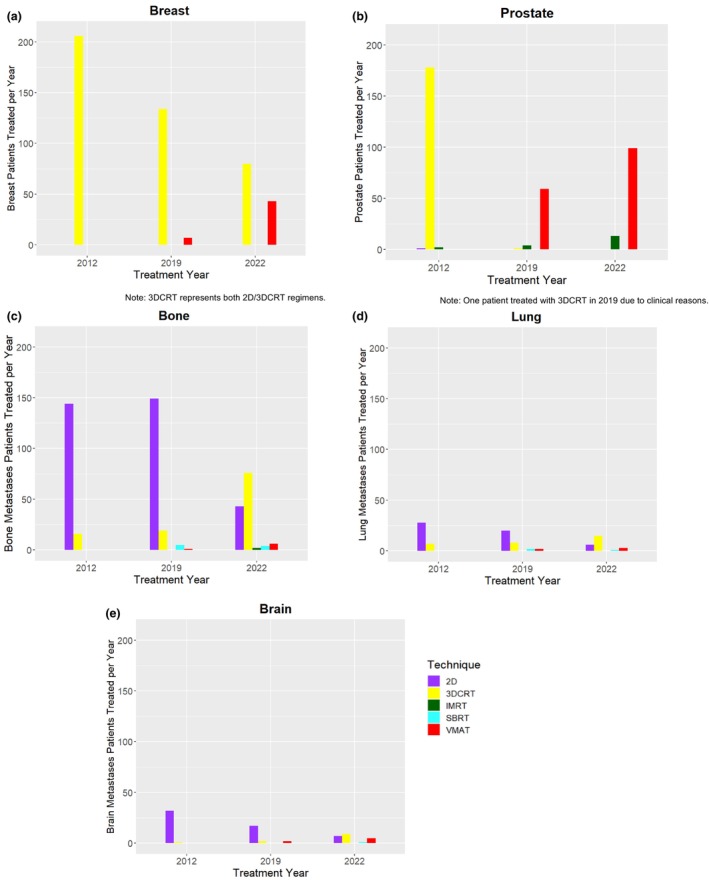
Relationship between treatment modality, treatment year and treatment site; breast (a), prostate (b), bone metastases (c), lung metastases (d) and brain metastases (e). 2D, 2‐dimensional field/undefined; 3DCRT, 3‐dimensional conventional radiation therapy; IMRT, intensity modulated radiation therapy; SBRT, stereotactic body radiation therapy; VMAT, volumetric modulated arc therapy.

The change in complexity for different treatment sites was analysed (Figure 6) and displayed that the majority of breast patients were being treated with 2‐dimensional and 3‐dimensional conformal radiation therapy (2D/3DCRT). 100% of whom were treated with this modality in 2012, 95% in 2019 and 65% in 2022, with the other 35% in 2022 being treated with volumetric modulated arc therapy (VMAT). For prostate patients, 98% of patients in 2012 were treated with 3DCRT. In 2022, 88.4% were treated with VMAT and 11.6% were treated with intensity modulated radiation therapy (IMRT). For palliative treatment sites, there was still a favourable trend towards static techniques, with all sites shifting from 2D to 3DCRT in 2022. In 2012, 80% of bone metastases patients were treated with 2D regimens and 20% with 3DCRT. In 2022, this shifted, with 32.8% treated with 2D, 58% with 3DCRT, 1.5% with IMRT, 4.6% with VMAT and 3.1% with stereotactic body radiation therapy (SBRT). For lung metastases patients, the shift from 2D to 3DCRT was similar, as the utilisation of 2D decreased from 80% in 2012 to 24% in 2022. In 2022, the other techniques included 3DCRT (60%), VMAT (12%) and SBRT (4%). Lastly, brain metastases patients had a similar trend, with 96% of patients treated with 2D in 2012 decreasing to 80.9% in 2019 and 31.8% in 2022. In 2022, 40.9% were treated with 3DCRT, 22.7% with VMAT and 4.5% with SBRT. With each site, the protocol for simulation, planning and treatment delivery increased in complexity (see Figure S2).

## 4 Discussion

This study found that while there has been an uptake of HFRT regimens for both curative and palliative treatment intents and most treatment sites, there was no clear trend with regard to social categories or patient characteristics at this single centre. Despite a decrease in the number of treatment fractions, the overall treatment complexity has increased.

The adoption of HF treatments for breast cancers in this study was similar to the data presented in previous literature. In 2020 and 2022, 95.6% of breast cancer prescriptions at two departments in New South Wales were HFRT [22]. In this study, 92.1% of breast cancer patients in 2022 were treated with HFRT radiotherapy and 5.5% with UFRT radiotherapy. As assessments of the adequacy of 26 Gy in 5# were only published recently, the uptake of UFRT regimens is expected to increase for breast cancer patients [1].

At TCC, the HFRT protocol was introduced for prostate patients during 2019, with 15.6% of patients treated with HFRT, and in 2022, 74.1%. Previous literature from New South Wales corroborates this, with no patients treated with HFRT in 2016, and 42% in 2019. With 23.2% of prostate cancer patients treated with CFRT in 2022, this may be because most published guidelines that supported the use of HFRT did not include trials involving pelvic lymph node treatment [23]. The small portion of patients treated with UFRT on the magnetic resonance linear accelerator (MRL) in 2022 correlates with the publications of stereotactic trials (PACE‐B) for prostate cancer [22].

For palliative patients, our data demonstrated a decrease in CFRT treatments and an increase in both HFRT and UFRT treatments. A retrospective review for Queensland cancer patients receiving palliative RT found that in 2017, 18% were prescribed single fractions, 42% were prescribed 2–5 fractions, 27% were prescribed 6–10 fractions and 13% were treated with more than 10 fractions [7]. Bone metastases showed a shift to UFRT regimens, with 84.8% in 2022, with 89% of those being single‐fraction treatments. From 2020 to 2022, 44.93% of bone metastases prescriptions were single fraction [22]. Similarly, with lung metastases, approximately a third of patients were treated with UFRT, with 35.9% in 2019 and 30.8% in 2022. With the feasibility of stereotactic and VMAT radiotherapy for lung metastases, this is expected. Brain metastases prescriptions had a different trend, with patients treated with UFRT remaining consistent, and HFRT treatments having a significant increase from 2012 and 2019 (56.8%, 56.5%) to 2022 (81.8%). In a retrospective study at a single institution, for patients treated with whole brain RT in 2005 to 2017, 86% were prescribed 30 Gy in 10# and 13% were prescribed 20 Gy in 5% [24]. This correlates with this study, as the adoption of 20 Gy in 5# was most prevalent in 2022.

This study was interested in assessing any relationships between fractionation type and specific patient characteristics. Transport costs during RT for CFRT patients have been reported to be more than double those of HFRT [25]. In this study, the number of patients travelling greater than 500 km to treatment significantly decreased after 2012, correlating with a private radiation oncology centre opening within the larger catchment area in early 2018, also contributing to the reduction in overall patient numbers treated in 2019 and 2022. In NSW, patients who travelled more than 150 km to their nearest radiation department were twice as likely to have HFRT [15]. Breast patients living regionally or remotely were 1.7 times more likely to receive HFRT radiotherapy than those who live in major cities [26]. We found no trend between distance to treatment and ARIA+ code with fractionation type, as all patient cohorts had an uptake of HFRT.

Trends between First Nations status and fractionation type were examined, as this has not been assessed in previous literature. No relationship was found in this data. For First Nations patients, RT can be difficult, as there is a loss of connection with culture when they are off Country [27]. Being away from home during treatment contributes to First Nations patients' compliance with treatment and holistic well‐being during treatment, which may contribute to significantly poorer cancer outcomes [28]. The utilisation of HFRT can offer less time away from country, their community and family.

Other than changes in fractionation in this study, there was a prominent shift to more conformal and modulated treatments in all assessed treatment sites (refer to Figure S2 for TCC treatment protocol changes, including modality and image guidance). For breast treatments, the data showed a small adoption of VMAT breast treatments. It is important to note that the data does not truly capture the increase in complexity, as additional workflow considerations were introduced in 2019 and 2022 for patients treated with 2D/3DCRT, including deep‐inspiration breath hold and hybrid IMRT planning. For prostate cancer patients, image‐guided VMAT treatments are the standard of care [26, 29]. At TCC, ultrasound intra‐fraction monitoring is utilised in patients treated for prostate only, introduced in 2016. In addition to this, some patients in 2022 were treated on the MRL, represented by the 13 patients treated with IMRT for prostate cancer in 2022. This does include not only initial treatment planning, but also daily replanning and online adaptive techniques, taking up to 60 min for a single treatment. While the breast and prostate treatments have reduced in number of fractions, the complexity and department resources required for these techniques in the simulation, planning and treatment process have arguably increased.

For bone, brain and lung metastases, there was an adoption of SBRT and modulated techniques, and a reduction of 3DCRT techniques in 2022. In 2012, all palliative sites were prominently treated with 2D fields such as an anterior–posterior opposed field arrangement, which can have a planning‐to‐treatment turnaround of 1 day. For whole brain treatments, historically, a manual calculation was performed; however, in 2019 and 2022 VMAT techniques were implemented for hippocampal sparing, optimal target coverage and organ at risk sparing [30]. One patient had stereotactic radiosurgery (SRS) in late 2022, corresponding with the implementation of an SRS service locally. For lung metastases, a small number of patients were treated with VMAT and SBRT in 2019 and 2022 and more patients treated with 3DCRT instead of 2D fields. While the data shows palliative treatment is still simple, 3DCRT has become increasingly complex compared to 10 years ago. A substantial portion of palliative patients was still treated with non‐modulated RT at TCC. For patients that have critical symptoms or are near the end of life, modulated RT may not provide any clinical benefit [31]. Less modulated treatment allows for a quicker turnaround and shorter appointments, which is beneficial for this patient cohort.

There were several limitations in this study. A large dataset meant manual corroboration of all extracted data was infeasible; however, particular cases were checked by the clinical investigators. Throughout the data cleaning, patients may have been inversly filtered out. Some patients may have been inadvertently removed where their treatment was delayed at the patient's request or other clinical reasons. Secondly, when re‐categorising the site of treatment for palliative patients, some patients may have been miscategorised. Due to the variability of treatment site descriptions between the radiation oncologists, patients that had a site description that did not fall into more routinely used site names were named ‘local control/advanced disease’. This is similar with the curative patients, as the ICD code was used to specify the treatment site; however, if the patient was being treated curatively for a secondary tumour, the ICD code extracted may not be an accurate representation of current treatment. Thirdly, further refinement of classifications of CFRT, HFRT and UFRT could be considered for future studies based on clinical protocol. Lastly, the extracted data lacked information of any additional specifics of the patients' treatments, such as deep‐inspiration breath hold, MRL, 6° of freedom (6‐DOF) and real time ultrasound imaging. This may have provided further insight into this study, particularly regarding the level of treatment complexity.

The sample size was large—a strength of this study; however, it is only sourced from a single department audit. Ideally, a comparison between departments in Queensland, metropolitan, regional, public and private, would be conducted to compare the adoption of various fractionation regimens and the use of complex treatments across different treatment sites and patient characteristics. In future contributions to this area of study, complexity changes could be more robustly researched, particularly to further inform resource and departmental management decisions. Conducting collaborative studies involving multiple departments and incorporating a qualitative analysis of physician decision‐making could improve our understanding of the underlying factors influencing treatment choices and fractionation. This study only included patient data where a treatment course was completed—future studies could analyse whether there is a relationship that exists between fractionation regimens and incomplete treatments, especially for those patients who live a significant distance from treatment centres.

The evident increase in HFRT from 2012 to 2022 within our data demonstrates value‐based health care provision for our population, as it reduces appointments and associated disruptions while maintaining clinical outcomes through evidence‐based practice fractionation schedules [32]. This has benefits for all patients, including limiting time away from work/carer duties, reducing travel and accommodation, particularly for those patients living out of town, and it benefits elderly patients who may be more frail [15, 33].

## 5 Conclusion

Overall, there was an increase in the utilisation of HF at the three timepoints over that decade (2012, 2019, 2022). Patient demographics, such as distance from treatment facilities and First Nations patients, did not significantly impact the prescription of HFRT. While the number of fractions for all treatment sites decreased, the overall workload and resources have escalated with increasing complexity of radiotherapy treatments. These findings provide valuable insights into the current practices surrounding HFRT radiotherapy, indicating that decision‐making by radiation oncologists may be primarily driven by clinical factors rather than patient demographics or social situations. The findings highlight the importance of evidence‐based practice, emphasising areas for further investigation to optimise treatment decision‐making and promote equitable access to HFRT treatments for all patients.

## Conflicts of Interest

The authors declare no conflicts of interest.

## Supporting information


Appendix S1.

## Data Availability

Data sharing is not available for this study due to ethical considerations.
