# Supplementary material for: Hypofractionation Utilisation in Radiation Therapy: A Regional Department Evaluation
Source: J Med Radiat Sci. 2025 Feb 25;72(Suppl 2):S31–41. doi: 10.1002/jmrs.857 (PMC12449595; doi:10.1002/jmrs.857)
Supplement: Supplementary file 1 — Appendix S1. [file JMRS-72-S31-s001.docx]

Supplementary Materials

Manuscript: Hypofractionation utilisation in radiation therapy: a regional department evaluation

### Table S1

Categorisation of SBRT treatment techniques in data analysis

| **Technique was changed to SABR if the following conditions were met** | Site Name | Technique |
| --- | --- | --- |
|  | Includes SABR | Stereo |
|  | Includes SABR | VMAT |
|  | Includes SABR | IMRT |
| All techniques named as SABR and Stereo was changed to SBRT for data analysis. | | |

Where SABR = stereotactic ablative body radiotherapy, stereo = stereotactic, VMAT = volumetric modulated arc therapy, IMRT = intensity modulated radiotherapy and SBRT = stereotactic body radiotherapy

### Table S2

Fractionation Schedules at TCC for Specific Treatment Sites

| **Cancer Site** | **CF** | | **HF** | | **UF** | |
| --- | --- | --- | --- | --- | --- | --- |
|  | **No. of #** | **Dose/# (Gy)** | **No. of #** | **Dose/# (Gy)** | **No. of #** | **Dose/# (Gy)** |
| *Curative* | ---- | <2.45 | ---- | <5 | ---- | $\geq$5 |
| *Breast* | 25 | 2 | 15 | 2.67 | 5 | 5 |
| *Prostate* | 39 | 2 | 20 | 3 | 7 | 6.1 |
| *Bone met.* | 10 | 3 | 5 | 4 | 1 | 8 |
| *Brain met.* | 10 | 3 | 5 | 4 | 5 | 6 |
| *Lung met.* | 10 | 3 | 5 | 4 | 1 | 8 |
| This is a generalisation of the prescriptions at TCC and may vary slightly with individual clinical considerations.  Where CF=conventionally fractionated, HF = hypofractionated, UF = ultra-fractionated, # = fractions and met = metastases Table S3 Breakdown of Data Set Characteristics   \| **Treatment Year** \| \| **2012** \| **2019** \| **2022** \| \| --- \| --- \| --- \| --- \| --- \| \|  \| \| Total Number of Patients \| \| \| \| *Treatment Site* \| *Breast* \| 252 \| 142 \| 126 \| \| *Prostate* \| 181 \| 64 \| 112 \| \| *Bone Mets.* \| 172 \| 183 \| 132 \| \| *Brain Mets.* \| 37 \| 23 \| 22 \| \| *Lung Mets.* \| 39 \| 39 \| 26 \| \| *Gender* \| *Males* \| 585 \| 411 \| 463 \| \| *Females* \| 475 \| 367 \| 327 \| \|  \| \| Age \| \| \| \| Age \| *(median)* \| 65 \| 67 \| 68 \| | | | | | | |

### Figure S1


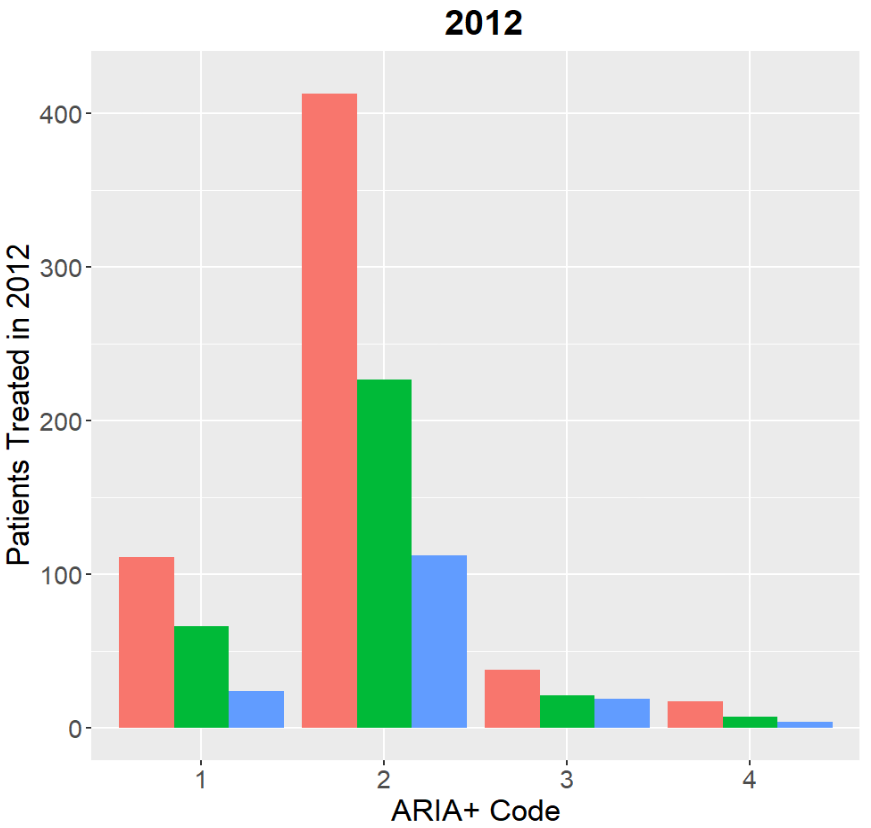

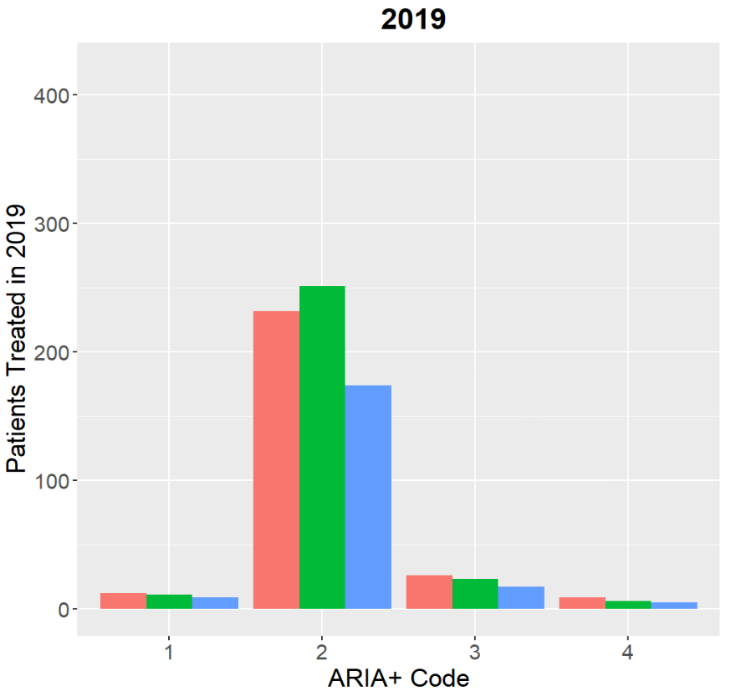
Relationship between ARIA+ codes, fractionation types and treatment years (a)2012, (b)2019 and (c)2022


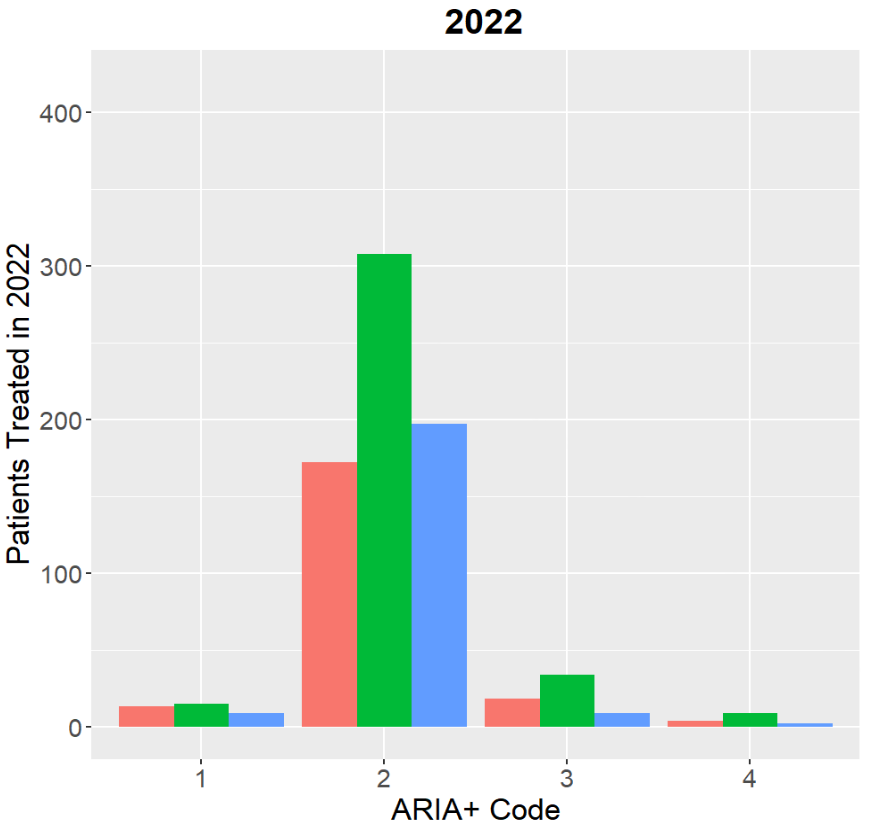

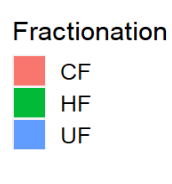


Where CF=conventional fractionation, HF=hypofractionation and UF=ultra-fractionation. Where 1 = inner regional, 2 = outer regional, 3 = remote and 4 = very remote

### Figure S2

TCC Protocol changes for (a)breast, (b)prostate and (c)palliative

CT Simulation

3D Planning

Independent Check

Treatment Delivery

Breast 2012

+ Delivered QA for IMRT/VMAT

+ Daily CBCT + DIBH for LHS

CT Simulation

VMAT Planning

Independent Check

Treatment Delivery

Breast 2022

+ DIBH coaching for LHS

Delivered QA for IMRT/VMAT

+ Daily CBCT + DIBH for LHS

CT Simulation

IMRT or VMAT Planning

Independent Check

Treatment Delivery

Breast 2019

+ Deep inspiration breath hold (DIBH) coaching for LHS

+ Maximum of weekly planar image verification

+ 4D CT for IMN

CT Simulation

3D Planning

Independent Check

Treatment Delivery

Prostate 2012

+ Delivered QA for IMRT/VMAT

+ Clarity US intrafraction monitoring

CT Simulation

IMRT or VMAT Planning

Independent Check

Treatment Delivery

Prostate 2019 & 2022

+ Clarity US sim

+ Clarity planning workflow

+ Gold Seed Insertion

+ Daily CBCT verification

Prostate MRL 2022

CT Simulation

IMRT Planning

Independent Check

Treatment Delivery

+ MRL Simulation

+ MRL planning workflow

+ Delivered QA for IMRT

+ Daily ATP/ATS replanning

+ MRL intrafraction monitoring

Post-Treatment Compiling of Treated Plans

CT Simulation

3D Planning

Independent Check

Treatment Delivery

Palliative 2012

CT Simulation

3D or VMAT planning

Independent Check

Treatment Delivery

Palliative 2019/2022

+ Delivered QA for IMRT/VMAT

+ Simulation with vacbag +/- compression

+ 4D CT for Chest patients

Palliative SABR 2019/2022

CT Simulation

DCAT or VMAT planning

Independent Check

+ Delivered QA for DCAT/VMAT

+ Separate SABR QA processes

Treatment Delivery

+ Multiple daily CBCT verification

+ Planar or CBCT verification

+ Planar image verification
